# Epitaxial Growth of Orthorhombic GaFeO_3_ Thin Films on SrTiO_3_ (111) Substrates by Simple Sol-Gel Method

**DOI:** 10.3390/ma12020254

**Published:** 2019-01-14

**Authors:** Minghui Zhang, Shintaro Yasui, Tsukasa Katayama, Badari Narayana Rao, Haiqin Wen, Xiuhong Pan, Meibo Tang, Fei Ai, Mitsuru Itoh

**Affiliations:** 1 State Key Laboratory of High Performance Ceramics and Superfine Microstructure, Shanghai Institute of Ceramics, Chinese Academy of Sciences, Shanghai 200050, China; zhangminghui@mail.sic.ac.cn (M.Z.); hqwen@mail.sic.ac.cn (H.W.); xhpan@mail.sic.ac.cn (X.P.); mbtang@mail.sic.ac.cn (M.T.); 2 Laboratory for Materials and Structures, Tokyo Institute of Technology, Midori-ku, Yokohama 226-8503, Japan; yasui.s.aa@m.titech.ac.jp (S.Y.); badari.rao@gmail.com (B.N.R.); 3 Center of Materials Science and Optoelectronics Engineering, University of Chinese Academy of Sciences, Beijing 100049, China; 4 Department of Chemistry, The University of Tokyo, Bunkyo-ku, Tokyo 113-0033, Japan; katayama@chem.s.u-tokyo.ac.jp

**Keywords:** GaFeO_3_ film, epitaxial growth, sol-gel method, multi-domain structure, magnetic property

## Abstract

A Sol-gel method assisted with spin-coating has been successfully used to grow orthorhombic GaFeO_3_ epitaxial films on SrTiO_3_ (111) substrates for the first time. The film with *Pna2_1_* crystal structure has been grown along the c-axis. The rocking curve of (004) reflection shows that the Full-Width at Half-Maximum (FWHM) value could be determined to be 0.230°, indicating good single crystallinity and high quality. X-ray Φ scan reveals a three-fold symmetry of the substrate and a six-fold symmetry of the film, respectively. The in-plane domains rotate 60° from each other in the film. Uniform film with dense structure, columnar grains with similar grain size was obtained. The thickness of the film was evaluated to be ~170 nm. The roughness value (RMS) measured by AFM was 4.5 nm, revealing a flat film. The in-plane Magnetization versus Magnetic field (*M-H*) curve at 5 K performs a typical ferri- or ferromagnetic hysteresis loop with a saturated magnetization (*M*_s_) value of 136 emu/cm^3^. The Curie temperature could be determined to be 174 K. Compared to Pulsed Laser Deposition (PLD), the sol-gel method can prepare large area films with low cost. These new films show promising applications in multiferroic devices.

## 1. Introduction

As the demand of environment protection, materials performance, device size, and energy saving is becoming higher, more and more attention is paid to the development of new materials with two or more functions. Multiferroic materials possessing magnetic and electric properties simultaneously in the single phase, supply a favorable method to design new devices with high performance. With the coupling of these two properties, these materials show interesting physics such as ferroelectric properties that can be changed by magnetic fields and ferromagnetic properties that can be controlled by electric field [1,2,3,4]. Multiferroic materials can be applied in the development of new devices like sensors [5,6,7], transducers [8,9], second harmonic generation [10] and information storage [11,12,13]. However, most of multiferroic materials exhibit spontaneous polarization and magnetization only at low temperature [14,15]. This would limit the application of multiferroicity. Recently, much attention has been focused on orthorhombic Ga_x_Fe_2−x_O_3_ (GFO) thin films which can be regarded as promising candidate materials performing magnetic and ferroelectric properties simultaneously at room temperature [16,17,18,19]. GFO has a *Pna2_1_* crystal structure, which is different from perovskite. The magnetic and ferroelectric properties of thin films are associated with its structure. Thin films with epitaxial structure are very helpful to obtain good multiferroism. 

However, favorable substrates and deposition conditions are required in order to prepare epitaxial GFO films. By far, most GFO thin films with oriented structure have been prepared only by some expensive techniques such as Pulsed Laser Deposition (PLD) [20,21,22]. In a previous study, Katayama et al. prepared GFO epitaxial films by PLD and controlled the crystal-domain orientations by changing substrates [23]. Magnetic properties were obtained at room temperature. Zhong et al. researched room temperature multiferroic properties in epitaxial GFO films with tunable Fe concentrations prepared by a dual target PLD method [24]. Katayama et al. performed a systematical study on the effect of composition on the ferroelectric and magnetic properties of GFO epitaxial films grown by PLD [1]. Therefore, GFO epitaxial films are promising room temperature multiferroic materials which show wide application prospects. However, as far as we know, high quality GFO epitaxial films are grown only by expensive PLD. It’s very difficult to prepare large area thin films by PLD, which would limit the applications of materials. 

In order to improve the usability of GFO epitaxial films in novel devices, a low cost preparation method which can grow large area thin films should be developed. In such methods, metal-organics are often used and theoretical calculation has also been employed [25,26]. A sol-gel method based on solutions can achieve homogeneous composition, wide-range tunable stoichiometry, low temperature heat treatment, with simple equipment and processes, and give the ability to prepare large area films [27,28,29]. These merits can accelerate the application of thin films greatly. Mishra et al. synthesized GFO polycrystalline thin films by a sol-gel method and researched room temperature multiferroic properties [30]. The results revealed that a chemical solution method was advantageous in preparing GFO thin films. If GFO epitaxial film can be grown by a sol-gel method, the quality and multiferroic properties of thin films can be greatly improved. This is important for GFO films to be regarded as a powerful competitor in designing commercial multiferroic devices compared with well-known bismuth ferrite. To the best of our knowledge, GFO epitaxial films grown by a sol-gel method have not been reported so far. 

In this study, orthorhombic GFO epitaxial films have been grown on SrTiO_3_ (111) (STO) substrates by a sol-gel method together with a spin-coating technique. The c-axis-oriented growth and six-fold in-plane symmetry of the films have been confirmed. The structure of GFO epitaxial films obtained by the sol-gel method is the same as that grown by PLD. The surface morphology and magnetic properties have been analyzed. The possibility of growing GFO epitaxial films by a simple sol-gel method has been confirmed.

## 2. Experiments

Precursor mixed solution should be prepared according to the stoichiometry of GaFeO_3_. GaO_1.5_ EMOD materials for films (SYM-GAO3 of Kojundo Chemical Laboratory Co., LTD, Sakado, Saitama, Japan, 0.3 mol/L), FeO_1.5_ EMOD materials for films (SYM-FEO5 of Kojundo Chemical Laboratory Co., LTD, 0.5 mol/L), and diluent agent for coating (Kojundo Chemical Laboratory Co., LTD, agent A) were taken as the starting materials. These three solutions were mixed to obtain a 0.1 M GaFeO_3_ solution. Then, magnetic stiring was used to mix the solution thoroughly. Finally, a stable, clear, and homogeneous processor solution could be obtained. Moreover, a 0.5 μm syringe filter was employed to filter the solution to get rid of the possible undissolved compositions. 

STO (111) substrates were cleaned ultrasonically in a glass cleaner (Semico clean 56) for 10 min and then in distilled water for 5 min. The resulted substrates were cleaned ultrasonically in distilled water for 10 min again before the deposition. A nitrogen gun was used to blow and dry the substrates. Then the substrates were put on a hot plate at 150 °C for 5 min. The substrate was fixed on a spin coater and the solution was dropped on to the substrate. The film was deposited by spin coating at a rotation speed of 1500 rpm for 20 s. The wet film was then heated on the hot plate at 150 °C for 5 min to dry the film. The above process including spin coating and drying was repeated 20 times. The resulted film was then annealed at 900 °C for 1 h in the air to crystallize the film to get epitaxial phase. The above cycle including spin coating, dry, and anneal was repeated 5 times to prepare high-quality and dense films. Finally, the film with a size of 10 × 10 mm had been prepared. 

High-resolution X-Ray Diffraction (XRD) (Rigaku Smartlab instrument, Akishima, Tokyo, Japan) was employed to measure the crystal structure of GFO films by Cu-Kα1 radiation. The surface morphology and roughness was analyzed by Atomic Force Microscopy (AFM) (ASYLUM MFP-3D, Oxford Instruments Company, Bicester, Oxfordshire, UK). The surface and cross section of the film were coated with gold for SEM (Hitachi, S-4800, Chiyoda-ku, Tokyo, Japan) measurement. Finally, in-plane magnetic properties were characterized by a superconducting quantum interference device (MPMS XL of Quantum Design Company, Toshima-ku, Tokyo, Japan). 

## 3. Results and Discussion

**Figure 1:**
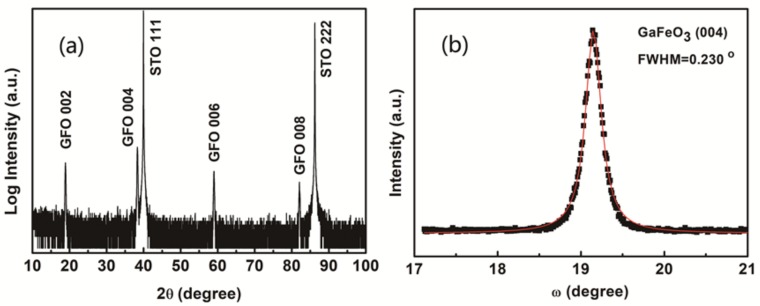
(**a**) Out of plane XRD 2θ-θ pattern of the GaFeO_3_ film grown on STO (111) substrate by sol-gel method and (**b**) the rocking curve of (004) reflection.

The high resolution XRD profile of the GFO film is presented in Figure 1a. There were four obvious diffraction peaks contributed by the films besides two peaks of STO substrates. The film diffraction peaks centered at around 18.9, 38.4, 59.1, and 82.1°. According to the previous research [19], those four peaks originated from (002), (004), (006), and (008) reflections of GFO films, respectively. It can be revealed that GFO (001) is parallel to STO (111) orientation in the grown GFO films. So out of plane XRD measurement indicates that the growth of the GFO films was along the c-axis direction. Furthermore, the spontaneous polarization is also along the c-axis in GFO [31]. This case was favorable to measure the ferroelectric properties of the films. The rocking curve of GFO films at (004) reflection was measured in Figure 1b. The black dots are experimental data, while the red solid line is the fitting curve. After fitting, the Full-Width at Half-Maximum (FWHM) value could be determined to be 0.230°. It can be concluded that good single crystallinity and high quality GFO films have been grown by the sol-gel method. 

**Figure 2:**
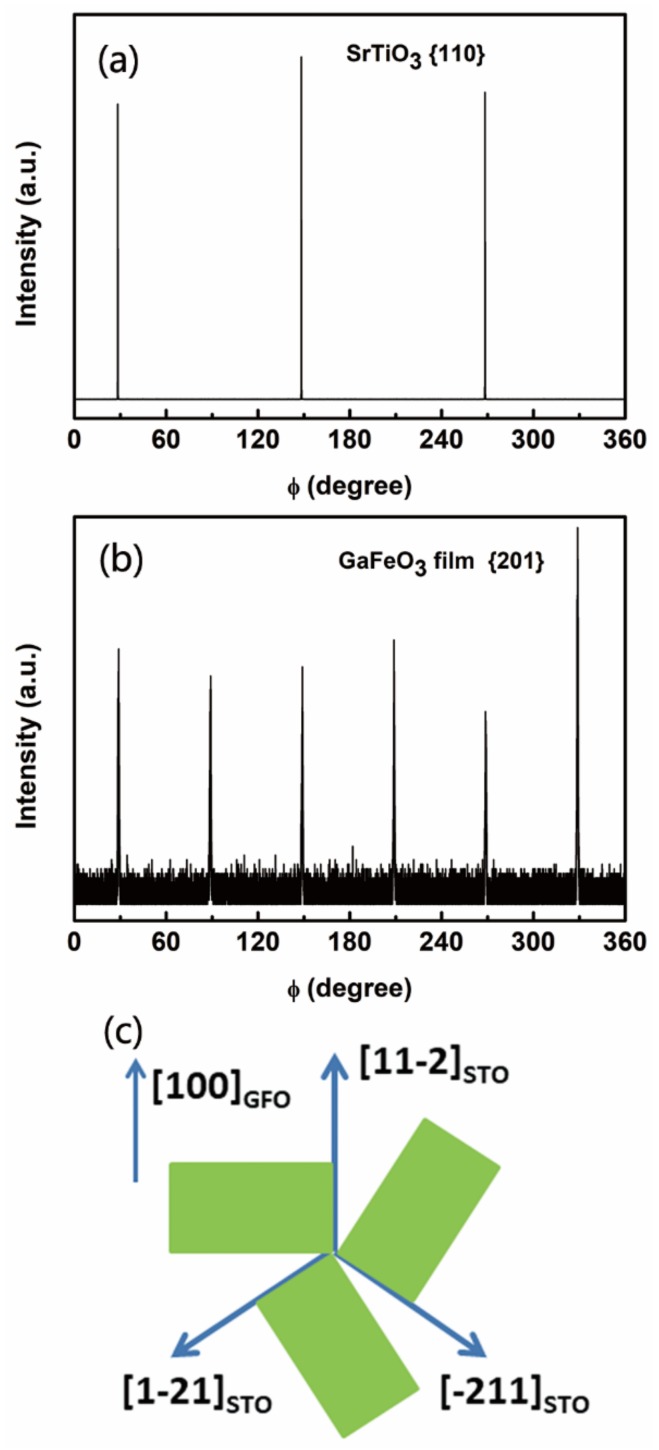
X-ray Φ scan patterns of (**a**) STO {110} substrate; (**b**) GFO {201} film, and (**c**) schematic presentation of the in-plane domain structure of GFO film on STO (111) substrate observed from the c-axis.

To reveal the in-plane orientation of the film, Φ scan of XRD was employed. The scan result around STO {110} reflections is presented in Figure 2a, indicating a three-fold symmetry of the substrate. Meanwhile, the scan result around GFO {201} reflections is presented in Figure 2b, showing a six-fold symmetry in the GFO film. So, multi-domain structure exists in the as-grown GFO films. Six sorts of in-plane domains appeared in the film as a result of the orthorhombic structure of GFO materials. Together with the Φ scan result, we can know that those in-plane domains rotate 60°from each other in the film. In another words, the in-plane domains could be divided into three groups each of which has two domains with opposite directions. So, three groups of in-plane domains rotate 120° from each other. Moreover, the projection of GFO {201} on the GFO (001) planes were parallel to the GFO [100] direction. And the projection of STO {110} on the STO (111) planes were parallel to the STO [11-2] direction [32]. Together with the three-fold symmetry of the STO (111) planes, the in-plane orientation can be thought as following: GFO [100] parallel to STO [11-2], GFO [100] parallel to STO [-211], and GFO [100] parallel to STO [1-21]. This relationship is described in Figure 2c. Therefore, c-axis oriented epitaxial GFO films are successfully obtained by the sol-gel technique which can prepare large area thin films with low cost compared to PLD. This starts a new road to accelerate the applications of novel multiferroic thin films in multifunctional devices.

**Figure 3:**
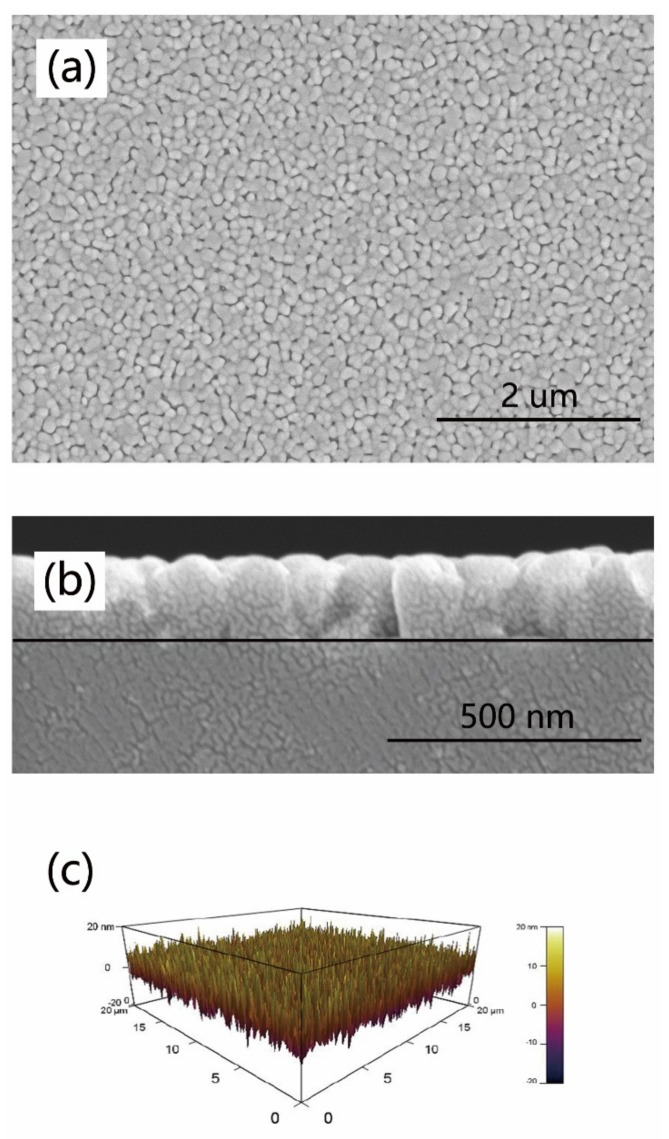
SEM images of (**a**) the surface and (**b**) cross-section of GFO films on STO (111) substrates. (**c**) 3-D AFM image of the film.

To evaluate the surface quality of GFO films, SEM and AFM were used to measure the samples. SEM images (Figure 3a) indicate that uniform film with unimodal grain size distribution has been deposited on the substrate. Moreover, the film showed a dense structure, indicating good quality. The cross sectional SEM micrograph is presented in Figure 3b. The grains had columnar shape, indicating that the crystals grew vertically from the substrate. The film had good orientation. According to the micrograph, the thickness of the thin film was evaluated to be ~170 nm. Surface morphology analysis of the GFO film is presented in Figure 3c in the form of 3-D by AFM. The scan area is 20 × 20 μm. AFM result demonstrates that the Root Mean Square (RMS) roughness value of the film was 4.5 nm. The roughness value of Ga_0.6_Fe_1.4_O_3_ film grown by PLD was 2.06 nm. And that of Al_0.2_Ga_0.4_Fe_1.4_O_3_ film by PLD was 8.73 nm [19]. The surface of the GFO film in the present study was flat and uniform, near to the level of the films prepared by PLD. Hence, sol-gel method can be employed to deposit new thin film with high-quality surface. 

**Figure 4:**
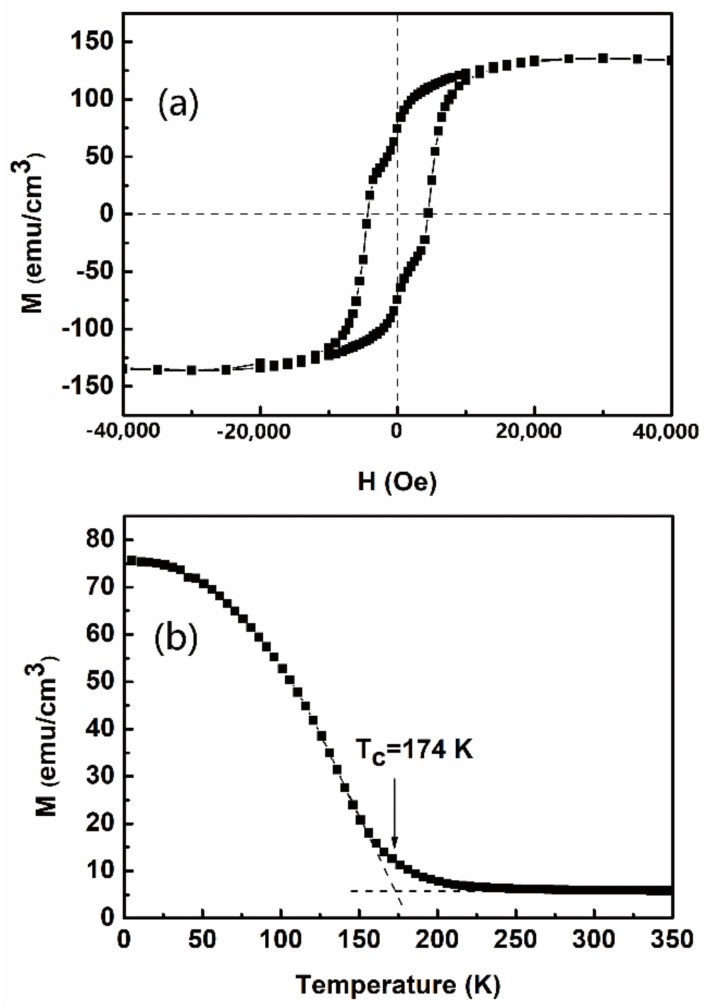
(**a**) The dependence of in-plane magnetization on magnetic field at 5 K, and (**b**) magnetization as a function of temperature at 500 Oe.

Easy magnetization direction of the GFO epitaxial films was along the a-axis. This axis is the in-plane direction. In order to study the magnetic properties, the in-plane Magnetization versus Magnetic field (*M-H*) curve of the GFO film was measured at 5 K. The diamagnetic contribution of the STO substrate was subtracted. Based on the surface area of the GFO film and the film thickness measured by cross section analysis of SEM, the film volume could be estimated by multiplying the above factors. Then, the magnetization values could be calculated by experimental data divided by the film volume. Finally, the result is shown in Figure 4a. The curve performs a typical ferri- or ferromagnetic hysteresis loop. The magnetization can achieve saturation at 25 kOe with a value of saturating magnetization (*M*_s_) of 136 emu/cm^3^ at 5 K. Figure 4b presents the dependence of field-cooled magnetization of the GFO film on temperature (*M-T*) measured by the application of a weak magnetic field of 500 Oe. The GFO film is ferrimagnetic in nature. According to Curie-Weiss law, two tangent lines are plotted in the *M-T* curve. And the Curie temperature (T_c_) could be evaluated to be 174 K. The T_c_ value of GFO epitaxial film grown by PLD is 200 K [1]. So the T_c_ value of the film prepared by the sol-gel method is close to that by PLD. Ga/Fe ratio in the film should be tuned by sol-gel method to improve the T_c_ value in future. 

## 4. Conclusions

GaFeO_3_ epitaxial films with a c-axis orientation on SrTiO_3_ (111) substrates have been grown by a simple sol-gel method with the help of spin-coating for the first time so far. High resolution XRD has been employed to measure the structure of the as-grown GFO film. Four diffraction peaks of the film are attributed to (002), (004), (006), and (008). This confirms that the film grew along the c-axis, which is the spontaneous polarization direction. The rocking curve of (004) reflection has been obtained, indicating that the FWHM value was estimated to be 0.230°. GFO films grown by the sol-gel method had good single crystallinity and high quality. The X-ray Φ scan was used to measure around STO {110} and GFO {201} reflections. The results showed a three-fold symmetry of the substrate and a six-fold symmetry of the film, respectively. The in-plane domains rotated 60° from each other in the film. In short, well-orientated epitaxial GFO films have been successfully grown by the sol-gel method. SEM results indicate that a uniform film with dense structure and similar grain size had been deposited on the substrate. Columnar grown gains with good orientation have been observed by cross sectional SEM. And the thickness of the film was evaluated to be ~170 nm. The AFM result revealed that the RMS roughness value was 4.5 nm, approaching the value of the films prepared by PLD. The film in the present study was flat. The in-plane *M-H* curve of the GFO film was measured at 5 K, displaying a typical ferri- or ferromagnetic hysteresis loop. The magnetization saturated at 25 kOe with a *M*_s_ value of 136 emu/cm^3^. The field-cooled *M-T* curve showed the value of T_c_ was evaluated to be 174 K, close to that by PLD. The GFO film performed ferrimagnetic behaviors. As an initial step, the sol-gel method has been successfully employed to grow GaFeO_3_ epitaxial films the quality of which approached that of films prepared by PLD. In future, it’s important to tune the composition of GaFeO_3_ films by the sol-gel method to achieve room-temperature multiferroic properties. The dependency of structure and properties of the films on deposition conditions is also worth being studied. Moreover, this simple method could also be used to grow new meta-stable films such as AlFeO_3_, ε-Fe_2_O_3_. The sol-gel method, which can prepare large size films, is simple and cheap. This is helpful for the development of new meta-stable films. The present study opens the door to accelerate the application of meta-stable multiferroic films in multifunctional devices.
